# Characteristics, outcome, duration of hospitalization, and cycle threshold of patients with COVID-19 referred to four hospitals in Babol City: a multicenter retrospective observational study on the fourth, fifth, and sixth waves

**DOI:** 10.1186/s12879-023-08939-w

**Published:** 2024-01-06

**Authors:** Farzin Sadeghi, Mehrdad Halaji, Hoda Shirafkan, Abazar Pournajaf, Hossein Ghorbani, Sara Babazadeh, Nafiseh Ezami, Kobra Fallhpour, Fatemeh Fakhraie, Shahrbano Gorjinejad, Saghar Saber Amoli, Fatemeh Hejazi Amiri, Mahnaz Baghershiroodi, Zahra Ahmadnia, Maryam Salehi, Mehdi Tourani, Jalal Jafarzadeh, Farzane Shanehbandpour Tabari, Seyed Raheleh Ahmadian, Rouzbeh Mohammadi Abandansari, Farzaneh Jafarian, Samaneh Rouhi, Arezoo Zabihollahi, Sarina Mostafanezhad, Fatemeh Saeedi, Arefeh Ebrahimian, Zeinab Deldar, Mahmoud Sadeghi Haddad Zavareh, Masoumeh Bayani, Mana Bazi Broun, Moein Shirzad, Siamak Sabbaghi, Mohsen Mohammadi, Rabeae Rahmani, Yousef Yahyapour

**Affiliations:** 1 Cellular and Molecular Biology Research Center, Health Research Institute, Babol University of Medical Sciences, Babol, Iran; 2 Infectious Diseases and Tropical Medicine Research Center, Health Research Institute, Babol University of Medical Sciences, Babol, Iran; 3 Social Determinants of Health Research Center, Health Research Institute, Babol University of Medical Science, Babol, Iran; 4 Clinical Research Development Unit of Rouhani Hospital, Babol University of Medical Sciences, Babol, Iran; 5 Department of Pathology, Ayatollah Rouhani Hospital, Babol University of Medical Sciences, Babol, Iran; 6 Part of Medical Records, Ayatollah Rouhani Hospital, Babol University of Medical Sciences, Babol, Iran; 7 Part of Infectious Control, Shahid Beheshti Hospital, Babol University of Medical Sciences, Babol, Iran; 8 Part of Infectious Control, Shahid Yahyanejad Hospital, Babol University of Medical Sciences, Babol, Iran; 9 Part of Infectious Control, Amirkola Hospital, Babol University of Medical Sciences, Babol, Iran; 10 Department of Medical Microbiology and Biotechnology Faculty of Medicine, Babol University of Medical Sciences, Babol, Iran; 11 Department of Medical Microbiology and Biotechnology Faculty of Medicine Guilan, University of Medical Sciences, City, Ondo, Nigeria; 12 Non-Communicable Pediatric Diseases Research Center, Health Research Institute, Babol University of Medical Sciences, Babol, Iran; 13 MSc. in Cellular and Molecular Biology, Education of Amol Teacher, Amol, Iran; 14 Biomedical and Microbial Advanced Technologies Research Center, Health Research Institute, Babol University of Medical Sciences, Babol, Iran

**Keywords:** COVID-19, SARS-CoV-2, Alpha variant, Delta variant, Omicron variant, Hospitalization, Death

## Abstract

**Background:**

The aim of the present study was to compare the epidemiological patterns of severe acute respiratory syndrome coronavirus 2 (SARS-CoV2) infections, hospitalizations, deaths, and duration of hospitalization during the fourth, fifth and sixth epidemic waves of coronavirus disease 2019 (COVID-19) in Iran.

**Methods:**

A multicenter retrospective observational study was conducted on hospitalized patients in four hospitals in the Babol district of northern Iran. The study periods were during the fourth, fifth, and sixth waves of the epidemic in Iran, (March 2021 to March 2022). A total of 13,312 patients with suspected COVID-19 were included. Patient demographics, medical history, length of hospital stay, and clinical outcomes were obtained from the hospital information system. Data on the cycle threshold (Ct) and SARS-CoV2 variant were collected for SARS-CoV2-positive cases.

**Results:**

The highest number of hospitalized patients was reported during the fifth (Delta) wave (5231; 39.3%), while the lowest number of hospitalized patients was reported during the sixth (Omicron) wave (2143; 16.1%). In total, 6459 (48.5%) out of 13,312 hospitalized patients with suspected COVID-19 had a positive rRT-PCR result. The fifth (Delta) wave had the highest number of SARS-CoV2 rRT-PCR-positive hospitalized patients (3573, 55.3%), while the sixth (Omicron) wave had the lowest number (835, 12.9%). Moreover, 238 (3.7%) patients with laboratory-confirmed COVID-19 died. The hospital mortality rate was 6.8% in the fourth (Alpha) wave, which reduced to 2.7 and 3.5% in the fifth (Delta) and sixth (Omicron) waves, respectively (*p* < 0.001).

**Conclusions:**

This is the most comprehensive study evaluating the epidemiologic characteristics of laboratory-confirmed SARS-CoV2 cases in Iran during the Alpha, Delta, and Omicron waves. The highest number of SARS-CoV2-positive hospitalized patients was in the fifth wave of COVID-19 (dominance of the Delta variant), while the sixth wave (dominance of the Omicron variant) had the lowest number. Comorbidities were similar, and cardiovascular disease, diabetes, kidney disease, and hypertension were the main risk factors in all waves.

## Background

In December 2019, the first case of severe acute respiratory syndrome coronavirus 2 (SARS-CoV-2) was identified in the city of Wuhan, Hubei, China, and the World Health Organization (WHO) declared a public health emergency and pandemic on 30 January and 11 March 2020, respectively [1, 2]. The global spread of the virus has been characterized by successive waves of infection with varying degrees of transmissibility and severity, which have been influenced by factors such as the emergence of different virus variants, the implementation of social distancing protocols, access to medical services, and vaccination efforts [3, 4].

As of March 20, 2023, more than 680 million cases and 6.8 million deaths have been reported worldwide, with Iran being one of the countries most affected by the virus in the Middle East. Since the first detection of SARS-CoV2 in Iran on February 19, 2020, the country has experienced eight waves of the pandemic. As of March 20, 2023, Iran had reported more than 7.5 million cases and 144,993 deaths due to the coronavirus disease 2019 (COVID-19).

The use of vaccines has helped to contain the disease, but the emergence of new variants of concern (VOCs), which have one or more of the following characteristics: high transmissibility, higher virulence, and lower efficacy of treatments or vaccines, has led to an increase in the disease burden [5, 6]. Throughout the pandemic, different variants of SARS-CoV-2 have been identified, some of which spread globally, while others quickly disappeared [7].

The first wave of infection was observed from February to May 2020, followed by the second wave from the end of June to September 2020. The third wave then occurred from October to December 2020. The fourth wave was triggered in early April 2021 and lasted until June 2021, while the fifth wave was detected from August to October 2021 [8]. In 2022, Iran experienced the sixth (January to the end of March) wave of the COVID-19 outbreak.

Studies have examined the length of hospitalization of people with COVID-19 and found differences in median length of stay in different countries [9].

According to a meta-analysis that evaluated 52 studies, COVID-19 patients had a median length of hospital stay of 14 days (with an interquartile range (IQR) of 10-19) in China and 5 days (with an IQR of 3-9) in the United Kingdom [10]. There are several factors that have been associated with prolonged hospitalization in COVID-19 patients. These include age, gender, the severity of COVID-19, the presence of comorbidities, and the ratio of patients to healthcare workers [11, 12].

Determining the length of hospital stay of individuals with COVID-19 can be beneficial for improving patient care, organizing the management of COVID-19 patients, and developing measures to shorten hospital stays, such as community care and patient follow-up. Nevertheless, existing data on the length of hospitalization of COVID-19 patients and associated factors in Iran are insufficient [13]. The aim of this study was to compare the epidemiological patterns of COVID-19 infections, hospitalizations, deaths, and duration of hospitalization during the fourth, fifth and sixth epidemic waves that occurred in Iran.

## Methods

### Study design and participants

This multicenter, retrospective observational study was conducted on hospitalized patients in four hospitals affiliated with Babol University of Medical Sciences (Ayatollah Rohani, Shahid Beheshti, Shahid Yahyanejad and Amirkola Children Hospital) during the fourth, fifth and sixth waves of the epidemic in Iran (March 2021 to March 2022).

The studied population included patients of all ages referred to four hospitals in Babol city who were suspected of COVID-19. The studied population was patients of all age groups with suspected COVID-19. The criteria for defining suspected COVID-19 cases were in line with those of the WHO [14]. SARS-CoV-2 infection was confirmed by real-time reverse transcription polymerase chain reaction (rRT-PCR) at the Molecular Diagnostic Reference Laboratory SARS-CoV-2 affiliated with Babol University of Medical Sciences. Demographic data such as age, gender, history of comorbidities, pregnancy, intensive care unit (ICU) admission, length of stay, and clinical outcomes (including recovery and death) were obtained from the hospital information system. The study included hospitalized patients with suspected COVID-19 and laboratory-confirmed SARS-CoV-2 infection status. Patients were excluded if they did not have an oropharyngeal and nasopharyngeal swab sample, had unclear rRT-PCR results, or their sample was analyzed with another diagnostic platform or at another facility.

#### Laboratory procedures

Samples from the oropharynx and nasopharynx were obtained from patients on admission using flocked swabs according to WHO established protocols [15].

All samples were sent to the Molecular Diagnostic Reference Laboratory for SARS-CoV-2 at Babol University of Medical Sciences for testing. Samples were handled according to standard laboratory biosafety protocols, without additional dilution or heat inactivation steps. Samples were then divided into small aliquots and stored at − 80 °C until analysis.

#### Viral nucleic acid extraction and rRT-PCR for SARS-CoV- 2

##### Detection and identification of affected variants

The Behperp Viral Nucleic Acid Extraction Kit (BehGene Biotechnology, Shiraz/Fars, Iran) was used to extract viral RNA according to the manufacturer’s instructions. Samples were rapidly analyzed using the GA SARS-CoV-2 OneStep RT-PCR Kit (GeneovA, Iran), a commercial triple-target assay for the detection of specific mutations in the S, N, and ORF1a genes of SARS-CoV-2.

Regarding the identification of VOCs, a good agreement between GA SARS-CoV-2 OneStep RT-PCR kit and identification of SARS-CoV-2 VOCs was observed in a recently published study [16]. Real-time RT-PCR was performed with QIAquant 96 5plex (Qiagen, Hilden, Germany).

Based on the cycle threshold (Ct) values obtained from the rRT-PCR analysis, the viral loads of the SARS-CoV-2 samples were evaluated comparatively.

Based on the diagnostic Ct values, the patients were divided into three groups: Group A with Ct values between 9 and 20; Group B with Ct values between 21 and 30; and Group C with Ct values between 31 and 40 [17].

#### Epidemic wave

A study of epidemiological characteristics was conducted in three epidemic waves, which occurred from March 25 to May 31, 2021(fourth wave-Alpha variant), from June 26 to December 1, 2021(fifth wave-Delta variant) and from January 17 to March 20, 2022 (sixth wave- Omicron variant). Patients who were referred between waves were also defined as a zero wave.

### Statistical analysis

SPSS 16 was used to analyze the data, with categorical variables summarized as frequencies and percentages. Chi-square and Fisher exact tests were used for comparison between groups and categories, respectively. Additionally, the two-sample z-test formula was used to evaluate the difference in proportions.

## Results

### Demographic status, comorbidity, and hospitalization of patients with suspected COVID-19

During the one-year study period from March 2021 to March 2022, 13,312 patients with suspected COVID-19 were admitted to hospitals in Babol City. The highest number of hospitalized patients was reported during the fifth (Delta) wave (5231; 39.3%), while the lowest number of hospitalized patients was reported during the sixth (Omicron) wave (2143; 16.1%). In addition, 3471 patients were reported between waves, as a zero wave (Table 1).

**Table 1:** Demographic characteristics, comorbidity status and outcomes in patients suspected with COVID-19 and SARS-CoV2 PCR-positive hospitalized patients

Characteristic	Total hospitalizations				*p*-value	SARS-CoV2 PCR Positive				*p*-value
	Wave 0^1^ (%)	4th (alpha) wave N (%)	5th (delta) wave N (%)	6th (omicron) wave N (%)		Wave 0 N (%)	4th (alpha) wave N (%)	5th (delta) wave N (%)	6th (omicron) wave N (%)	
Overall	3417 (25.5)	2519 (18.9)	5231 (39.3)	2143 (16.1)	–	644 (10)	1402 (21.7)	3573 (55.3)	835 (12.9)	–
Median age, yrs., (IQR)	56 (39)	56 (31)	52 (28)	62 (38)	< 0.001	55 (27)	53 (24)	52 (23)	65 (34)	< 0.001
Mean ± SD, age, yrs	49.66 ± 27.4	53.6 ± 21.82	49.5 ± 21.7	54.3 ± 27.5	< 0.001	54.7 ± 19.3	53.5 ± 16.9	51.1 ± 17.4	58.6 ± 25.1	< 0.001
Age group, yrs.										
< 18 (*n* = 1690; 12.8%)	634 (18.8)	185 (7.4)	522 (10)	349 (16.4)	< 0.001	23 (3.6)	18 (1.3)	121 (3.4)	84 (10.1)	< 0.001
18–49 (*n* = 3721; 28.1%)	776 (23)	781 (31)	1803 (34.6)	361 (16.9)		216 (33.6)	567 (40.4)	1445(40.5)	160 (19.2)	
50–64 (*n* = 3443; 26%)	737 (21.9)	686 (27.3)	1557 (29.9)	463 (21.7)		206 (32)	444 (31.7)	1215 (34)	166 (19.9)	
65-79 (*n* = 2908; 22%)	770 (22.9)	588 (23.4)	977 (18.7)	573 (26.9)		125 (19.4)	276 (19.7)	649 (18)	246 (29.5)	
≥ 80 (*n* = 1468; 11.1%)	451 (13.4)	276 (11)	353 (6.8)	388 (18.2)		73 (11.4)	97 (6.9)	149 (4.2)	177 (21.2)	
Sex										
Men	1754 (51.3)	1188 (47.2)	2392 (45.7)	1005 (46.9)	< 0.001	309 (48)	614 (43.8)	1533(42.9)	377 (45.1)	0.097
Women	1663 (48.7)	1331 (52.8)	2839 (54.3)	1138 (53.1)		335 (52)	788 (56.2)	2040(57.1)	458 (54.9)	
Underlying diseases										
CVD^2^	824 (24.1)	652 (25.9)	857 (16.4)	635 (29.6)	< 0.001	158 (24.5)	298 (21.3)	484 (13.5)	243 (29.1)	< 0.001
Diabetics	592 (17.3)	433 (17.2)	751 (14.4)	397 (18.5)	< 0.001	116 (18)	236 (16.8)	520 (14.6)	173 (20.7)	< 0.001
KD^3^	137 (4)	72 (2.9)	103 (2)	80 (3.7)	< 0.001	17 (2.6)	33 (2.4)	53 (1.5)	34 (4.1)	< 0.001
Hypertension	444 (13)	195 (7.7)	372 (7.1)	239 (11.2)	< 0.001	102 (15.8)	101 (7.2)	232 (6.5)	92 (11)	< 0.001
Malignancies	243 (7.1)	123 (4.9)	160 (3.1)	122 (5.7)	< 0.001	18 (2.8)	29 (2.1)	47 (1.3)	40 (4.8)	< 0.001
RD^4^	136 (4)	82 (3.3)	100 (1.9)	73 (3.4)	< 0.001	15 (2.3)	49 (3.5)	54 (1.5)	29 (3.5)	< 0.001
GID^5^	34 (1)	24 (0.95)	31 (0.62)	12 (0.54)	0.061	5 (0.8)	13 (0.9)	11 (0.33)	8 (0.9)	0.005
BND^6^	230 (6.7)	132 (5.2)	119 (3.4)	160 (7.5)	< 0.001	35 (5.4)	48 (3.4)	84 (2.4)	75 (9)	< 0.001
BD^7^	23 (0.7)	18 (0.7)	14 (0.3)	13 (0.6)	0.016	2 (0.3)	10 (0.7)	9 (0.3)	3 (0.4)	0.118
Pregnancy	57 (1.7)	19 (0.8)	73 (1.4)	30 (1.4)	0.024	25 (3)	8 (0.6)	54 (1.5)	25 (3)	0.001
Others^8^	25 (0.7)	31 (1.2)	29 (0.6)	21 (1)	0.012	3 (0.5)	19 (1.4)	12 (0.4)	11 (1.3)	< 0.001
Comorbidities										
No Comorbidities	1570 (45.9)	1219 (48.4)	3346 (64)	967 (45.1)	< 0.001	307 (47.7)	767 (57.7)	2458(68.8)	344 (41.2)	< 0.001
One	1187 (34.7)	917 (36.4)	1258 (24)	725 (33.8)		222 (34.5)	455 (32.5)	749 (21)	301 (36)	
> = Two	660 (19.3)	383 (15.2)	627 (12)	451 (21)		115 (17.9)	180 (12.8)	366 (10.2)	190 (22.8)	
Hospitalization outcome										
Length of stay, days, median (IQR)	5 (4)	5 (4)	5 (3)	5 (5)	< 0.001	5 (3)	5 (3)	5 (3)	5 (4)	0.066
Length of stay, days, Mean ± SD	7.6 ± 9.0	7.5 ± 11.3	7.1 ± 8.6	6.6 ± 6.0	< 0.001	7.03 ± 10.9	6.6 ± 9.4	6.7 ± 7.5	6.3 ± 5.7	0.744
ICU admission	139 (4.1)	97 (3.9)	176 (3.4)	75 (3.5)	0.344	49 (7.6)	62 (4.4)	138 (3.9)	42 (5)	< 0.001
In-hospital death	72 (2.1)	167 (6.6)	139 (2.7)	43 (2)	< 0.001	17 (2.6)	95 (6.8)	96 (2.7)	29 (3.5)	< 0.001

1) Wave 0: related to between waves of SARS-CoV-2 Infections; 2) CVD: Cardiovascular Diseases; 3) KD: Kidney Diseases; 4) RD: Respiratory Disorders; 5) GID: Gastrointestinal Diseases; 6) BND) Brain & Neurologic Diseases; 7) BD: Blood Disorders; 8) Others including: Special diseases, Thyroiditis, Lupus and Immunodeficiency diseases

**Fig. 1:**
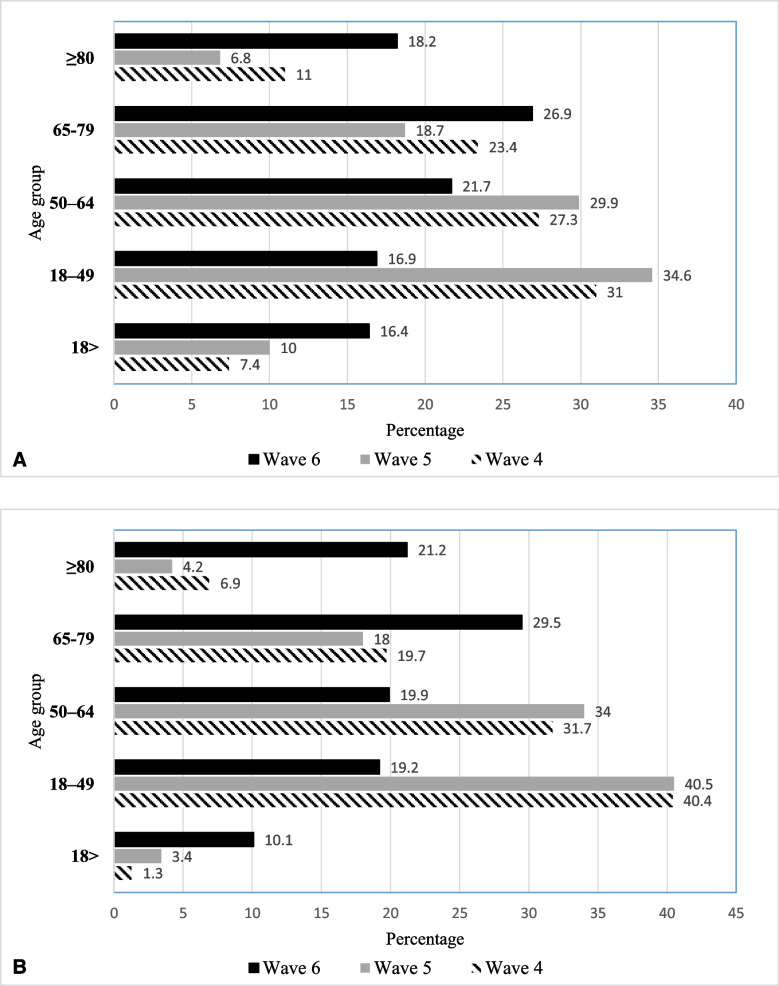
Age group distribution of hospitalized patients with suspected (**A**) and confirmed (**B**) COVID-19–infection

The mean age of suspected COVID-19 cases was 51.1 ± 24.4 years, 6972 (52.4%) were female, and 4376 (33.1%) were > 65 years. The highest hospitalization rates during the Alpha and Delta waves were reported in the 18-49-year-old age group, with rates of 31 and 34.6%, respectively. In contrast, most hospitalizations during the Omicron wave were reported in the age group of ≥65 years, with a rate of 45.1% (Table 1 and Fig. 1).

The data from the present study showed that 30.7% of patients with suspected comorbidity had one comorbidity, while 15.9% had two or more comorbidities. The frequency of each comorbidity ranged from 0.5 to 22.3%. In all waves, the two most common comorbidities were cardiovascular disease (CVD) (2968 patients [22.3%]) and diabetes (2173 patients [16.3%]) (Table 1). Four hundred and eighty-seven patients (3.7%) had to be treated in the ICU and 422 (3.2%) of the patients died. In addition, the percentage of patients requiring ICU decreased from 3.9% in the fourth wave to 3.5 and 3.4% in the sixth and fifth waves, respectively. The duration of hospitalization was 7.6 ± 9.0, 7.5 ± 11.3, 7.1 ± 8.6 and 6.6 ± 6.0 days for patients with suspected COVID-19 in the zero, fourth, fifth, and sixth waves, respectively. The data in Table 1 indicate that the average mortality rate among the participants was 3.2% (422 individuals). The fourth wave had the highest mortality rate of 6.6% (167 individuals), while the sixth wave had the lowest rate of 2% (43 individuals).

### Demographic status, comorbidity and hospitalization outcomes of SARS-CoV2 PCR-positive patients

In total, 6459 (48.5%) of 13,312 patients with suspected COVID-19 who were hospitalized had a positive rRT-PCR result. The demographic information, comorbidities, and outcomes of patients in the fourth, fifth, and sixth waves are compared in Table 1. The fifth wave had the highest number of hospitalized SARS-CoV-2 PCR-positive patients (3573, 55.3%), while the sixth wave had the lowest (835, 12.9%). Moreover, 644 (10%) patients were recorded in the zero wave (Table 1). The mean age of SARS-CoV-2 PCR-positive patients was 52.9 ± 18.8 years, 3621 (56.1%) were female and 2390 (37%) were over 18–49 years old. The characteristics of the patients are summarized in Table 1.

In addition, the proportion of men was 43.8% in the fourth wave and increased to 45.1% in the sixth wave. However, there was no significant difference in the prevalence of men in the waves (*p* = 0.097). In the fourth wave, the mean age was 53 years, in the fifth wave it decreased to 52 years, and in the sixth wave, it increased significantly to 65 years (*p* < 0.001).

In the fourth and fifth waves, the largest percentage of patients was between 18 and 49 years old, while in the six wave, the largest percentage of patients (50.7%) was > 65 years old (p < 0.001).

An analysis of the distribution of comorbidities revealed that CVD (18.2%), diabetes (16.2%), and hypertension (8.2%) were the most common comorbidities in patients with positive rRT-PCR results in all waves. Statistical analysis indicates that the distribution of comorbidities varied significantly between waves, with the exception of Blood Disorders (BD) (*p* = 0.118), as illustrated in Table 1.

The data in Table 1 illustrate that there is no statistically significant difference in the mean number of hospital days among patients hospitalized during the three COVID-19 waves (*p* = 0.744). Nevertheless, the highest mean number of hospitalization days was observed in the fifth wave (6.7 ± 7.5). Moreover, the proportion of patients transferred to the ICU increased significantly from 4.4% in the fourth wave to 5% in the sixth wave.

**Fig. 2:**
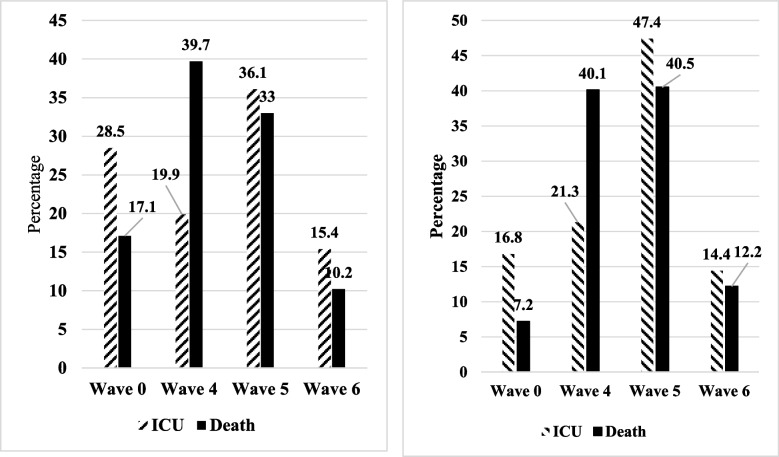
Percentage of patients admitted to the intensive care unit (ICU) and mortality rate among SARS-CoV-2 PCR-positive (**A**) and total hospitalizations (**B**) based on epidemic waves

During the study period, 238 (3.7%) patients with confirmed COVID-19 died. The percentage of confirmed COVID-19 patients who died in hospital was 6.8% in the fourth wave and decreased to 2.7 and 3.5% in the fifth and sixth waves, respectively (*p* < 0.001) (Table 2 and Fig. 2).

**Table 2:** Mortality and ICU admission status in COVID-19 suspected and SARS-CoV2 PCR-positive hospitalized patients

Characteristic	Total hospitalizations					SARS-CoV-2 PCR Positive				
	Total	ICU	*p*-value	Death	*p*-value	Total	ICU	*p*-value	Death	*p*-value
Overall	13,312	487	–	422	–	6459	291	–	238	–
Median age, yrs., (IQR)	55 (32)	62 (28)	< 0.001	66 (28)	< 0.001	54 (26)	62 (26)	< 0.001	65 (28)	< 0.001
Mean ± SD age, yrs	51.9 ± 24.4	57.9 ± 23.2	< 0.001	63.0 ± 20.8	< 0.001	52.9 ± 18.8	56.0 ± 21.0	< 0.001	52.5 ± 18.7	0.002
Age group, yrs										
< 18	1690 (12.8)	40 (8.2)	< 0.001	17 (4.1)	< 0.001	246 (3.8)	16 (5.5)	< 0.001	5 (2.1)	< 0.001
18–49	3723 (28.1)	100 (20.6)		74 (17.7)		2390 (37)	70 (24.1)		51 (21.4)	
50–64	3443 (26)	130 (26.7)		105 (25.2)		2032 (31.5)	82 (28.2)		62 (26.1)	
65-79	2908 (22)	143 (29.4)		119 (28.5)		1290 (20)	82 (28.2)		68 (28.6)	
≥ 80	1468 (11.1)	73 (15)		102 (24.5)		497 (7.7)	41 (14.1)		52 (21.8)	
Sex										
Men	6340 (47.6)	241 (49.5)	0.402	212 (50.2)	0.275	2833 (43.9)	135 (46.4)	0.373	118 (49.6)	0.07
Women	6972 (52.5)	246 (50.5)		210 (49.8)		3626 (56.1)	156 (53.6)		120 (50.4)	
Underlying diseases										
CVD^2^	2968 (22.3)	130 (26.7)	0.018	133 (31.5)	< 0.001	1184 (18.3)	79 (27.1)	< 0.001	61 (25.6)	0.003
Diabetics	2173 (16.3)	111 (22.8)	< 0.001	86 (20.4)	0.022	1046 (16.2)	65 (22.3)	0.004	51 (21.4)	0.026
KD^3^	394 (3)	22 (4.5)	0.039	27 (6.4)	< 0.001	137 (2.1)	13 (4.5)	0.004	14 (5.9)	< 0.001
Hypertension	1250 (9.4)	61 (12.5)	0.016	41 (9.7)	0.816	528 (8.2)	28 (9.6)	0.356	18 (7.6)	0.726
Malignancies	649 (4.9)	27 (5.5)	0.485	27 (6.4)	0.140	135 (2.1)	9 (3.1)	0.221	12 (5)	0.001
RD^4^	391 (2.9)	25 (5.1)	0.003	22 (5.2)	0.005	147 (2.3)	9 (3.1)	0.339	11 (4.6)	0.013
GID^5^	100 (0.75)	4 (0.82)	0.855	5 (1.2)	0.294	36 (0.55)	2 (0.7)	0.761	3 (1.3)	0.138
BND^6^	701 (5.3)	49 (10.1)	< 0.001	35 (8.3)	0.005	242 (3.7)	23 (7.9)	< 0.001	17 (7.1)	0.005
BD^7^	68 (0.5)	5 (1.02)	0.104	4 (0.94)	0.201	24 (0.4)	2 (0.7)	0.365	3 (1.3)	0.022
Pregnancy	179 (1.3)	2 (0.4)	0.680	1 (0.2)	0.045	94 (1.5)	2 (0.7)	0.263	1 (0.4)	0.174
Others^8a^	106 (0.72)	5 (1.02)	0.560	4 (0.94)	0.722	47 (0.72)	5 (1.7)	0.042	2 (0.8)	0.835
Comorbidities										
No Comorbidities	7103 (53.4)	192 (39.4)	< 0.001	150 (35.5)	< 0.001	3878 (60)	130 (44.7)	< 0.001	99 (41.6)	< 0.001
One	4088 (30.7)	185 (38)		185 (43.8)		1729 (26.8)	100 (34.4)		97 (40.8)	
> =Two	2121 (15.9)	110 (22.9)		87 (20.6)		852 (13.2)	61 (21)		42 (17.6)	
Waves of disease										
Wave 0	3417 (25.5)	139 (28.5)	0.344	72 (17.1)	< 0.001	646 (10)	49 (16.8)	< 0.001	17 (7.2)	< 0.001
4th (alpha) wave	2519 (18.9)	97 (19.9)		167 (39.7)		1402 (21.7)	62 (21.3)		95 (40.1)	
5th (delta) wave	5231 (39.3)	176 (36.1)		139 (33)		3574 (55.3)	132 (47.4)		96 (40.5)	
6th (omicron) wave	2143 (16.1)	75 (15.4)		43 (10.2)		836 (12.9)	42 (14.4)		19 (12.2)	
Hospitalization outcome^b^										
Length of stay, days, median (IQR)	5 (4)	11 (14)	< 0.001	7 (14)	< 0.001	5 (3)	11 (13)	< 0.001	5 (3)	< 0.001
Length of stay, days, Mean ± SD	7.2 ± 9.5	15.8 ± 17.8	< 0.001	12 ± 14.3	< 0.001	6.7 ± 8.1	14.2 ± 12.3	< 0.001	6.4 ± 7.9	< 0.001

^a^Including: Special diseases, Thyroiditis, Lupus and immunodeficiency diseases. ^b^823 cases were missed in terms of length of stay. So, Hospitalization outcome was calculated for 12,489 suspected COVID-19 patients

1) Wave 0: related to between waves of SARS-CoV-2 Infections; 2) CVD: Cardiovascular Diseases; 3) KD: Kidney Diseases; 4) RD: Respiratory Disorders; 5) GID: Gastrointestinal Diseases; 6) BND) Brain & Neurologic Diseases; 7) BD: Blood Disorders; 8) Others including: Special diseases, Thyroiditis, Lupus and Immunodeficiency diseases

### The rRT-PCR cycle threshold values

According to Table 3, the Ct values of positive rRT-PCR tests were divided into three groups: 9-20 (1695; 26.6%), 21-30 (3879; 60.8%), and 31-40 (805; 12.6%). Statistical analysis showed that the Ct groups differed significantly in terms of kidney disease (KD) (*p* = 0.011) and pregnancy (0.009). Mortality rates differed significantly between the three Ct groups, with the highest rate (6.1%) observed in patients with Ct values between 9 and 20 (p < 0.001). Additionally, patients with lower Ct values (higher viral load) were admitted to the ICU more frequently. Furthermore, 5.3% of patients with Ct values between 9 and 20 compared to 3% of patients with Ct values between 31 and 40 had to be admitted to the ICU. Table 3 represents the length of stay for each Ct group, and no significant difference is found in the average length of stay between the Ct groups.

**Table 3:** Demographic characteristics and outcomes in SARS-CoV2 PCR-positive hospitalized patients based on cycle threshold (Ct) range

Characteristic	Total	Ct value range			*p*-value
		9 - 20	21 - 30	31 - 40	
Overall	**6379**	**1695**	**3879**	**805**	**–**
Median age, yrs., (IQR)	54 (26)	55 (27)	53 (25)	54 (25)	0.066
Mean ± SD age, yrs	52.93 ± 18.81	53.03 ± 20.5	52.9 ± 17.9	52.6 ± 19.6	0.744
Age group, yrs					
< 18	245 (3.8)	101 (6)	101 (2.6)	43 (5.3)	< 0.001
18–49	2363 (37.1)	560 (33.1)	1531 (39.5)	272 (23.8)	
50–64	2003 (31.4)	528 (31.2)	1208 (31.2)	267 (33.2)	
65-79	1277 (20)	346 (20.4)	774 (20)	157 (19.5)	
≥ 80	487 (7.6)	159 (9.4)	262 (6.8)	66 (8.2)	
Sex^a^					
Men	2769 (43.8)	734 (43.3)	1681 (43.3)	381 (47.3)	0.101
Women	3583 (56.2)	961 (56.7)	2198 (56.7)	424 (52.7)	
Underlying diseases					
CVD	1162 (18.2)	313 (18.5)	685 (17.7)	164 (20.4)	0.183
Diabetics	1032 (16.2)	287 (16.9)	600 (15.5)	145 (18)	0.126
Kidney diseases	135 (2.1)	51 (3)	68 (1.8)	16 (2)	0.011
Hypertension	524 (8.2)	119 (7)	337 (8.7)	68 (8.4)	0.11
Malignancies	135 (2.1)	39 (2.3)	75 (1.9)	21 (2.6)	0.397
RD	146 (2.3)	39 (2.3)	88 (2.3)	19 (2.4)	0.987
Liver diseases	33 (0.5)	8 (0.5)	23 (0.6)	2 (0.2)	0.443
GID	4 (0.06)	1 (0.06)	3 (0.1)	0 (0.0)	0.726
BND	240 (3.4)	72 (4.2)	140 (3.6)	28 (3.5)	0.464
BD	24 (0.4)	7 (0.4)	15 (0.4)	2 (0.2)	0.809
Pregnancy	94 (1.5)	38 (2.2)	47 (1.2)	9 (1.1)	0.009
Others	47 (0.7)	11 (0.6)	27 (0.7)	9 (1.1)	0.393
Comorbidities					
No-Comorbidities	3834 (60.1)	983 (58)	2394 (61.7)	457 (56.8)	0.009
One	1701 (26.7)	492 (29)	977 (25.2)	232 (28.8)	
≥ Two	844 (13.2)	220 (13)	508 (13.1)	116 (14.4)	
Hospitalization outcome					
Length of stay, days, median (IQR)	5 (3)	5 (3)	5 (3)	5 (3)	0.054
Length of stay, days, Mean ± SD	6.7 ± 8.1	6.8 ± 7.01	6.60 ± 7.85	6.69 ± 11.39	0.054
ICU admission	289 (4.5)	90 (5.3)	175 (4.5)	24 (3)	0.33
Death	235 (307)	103 (6.1)	103 (2.7)	29 (3.6)	< 0.001
SARS-CoV-2 epidemic waves and variant predominance status					
Waves 0	633 (9.9)	106 (6.3)	427 (11)	100 (12.4)	< 0.001
4th (alpha) wave	1355 (21.2)	444 (26.2)	742 (19.1)	169 (21)	
5th (delta) wave	3554 (55.7)	919 (54.2)	2207 (56.9)	428 (53.2)	
6th (omicron) wave	836 (13.1)	226 (13.3)	502 (12.9)	108 (13.4)	

1) Wave 0: related to between waves of SARS-CoV-2 Infections; 2) CVD: Cardiovascular Diseases; 3) KD: Kidney Diseases; 4) RD: Respiratory Disorders; 5) GID: Gastrointestinal Diseases; 6) BND) Brain & Neurologic Diseases; 7) BD: Blood Disorders; 8) Others including: Special diseases, Thyroiditis, Lupus and Immunodeficiency diseases

^a^27 cases were missing

## Discussion

This retrospective cross-sectional study investigated the data of 13,312 patients with suspected COVID-19 who were admitted to teaching hospitals in Babol district during three COVID-19 epidemic waves in northern Iran. The aim of the present observational study was to compare the impact of three different consecutive COVID-19 epidemic waves (each wave with a different SARS-CoV-2 variant) on mortality, ICU admission, and hospitalization.

Thus, the current study investigated three COVID-19 epidemic waves of hospitalizations between March 2021 and March 2022, each related to the Alpha, Delta, and Omicron variants of SARS-CoV-2. In March 2021, the fourth wave of COVID-19 hospitalizations occurred in northern Iran, followed by the fifth wave in June 2021 and the sixth wave in January 2022. Our experience with the fourth, fifth and sixth waves was different from the previous waves in northern Iran. In the latter three waves, the number of male hospitalizations and deaths was much lower, while the time of bed occupancy in the hospital decreased [18].

In the present study, the majority of patients in the fifth epidemic wave were affected by the Delta variant (55.3%). There is evidence that the replication rate of the Delta variant is much faster than that of the Alpha variant and that the Delta variant is more contagious compared to the original SARS-CoV-2 [19]. According to one study, the virus concentration in infections with the Delta variant was 1000 times higher than with other variants [15]. Based on this report, the Delta variant was declared the “fastest and fittest” variant of SARS-CoV-2 by the WHO [20].

In the fourth and fifth waves, the proportion of patients aged 18–49 was significantly higher than in the sixth wave. However, the proportion of patients aged ≥65 years was significantly higher in the sixth wave. According to the results of the ongoing study, the patients who dealt with COVID-19 in the sixth wave were generally older people (with the mean age of 58.6 years). In the fourth and fifth waves, however, it was mainly young people who were affected (Mean age of 53.5 and 51.1 years, respectively).

The present study found a demographic shift towards more women and younger people affected by the fourth to sixth wave of the COVID-19 epidemic. This is in line with other reports on the demographic shift of the pandemic in other countries [21–23].

Mousavi et al. explain, in partial agreement with our findings, that patients affected by COVID-19 in the fifth wave were younger than in the third and fourth waves in Iran [24]. Moreover, Zali et al. reported that in the fifth (delta) wave, the proportion of patients aged 60 years was significantly high, while the number of patients aged ≥60 years was significantly lower [25].

Another important finding of the current study is that the patients in the waves were more likely to be female. However, no significant differences in positive rRT-PCR results were found between women and men (*p* = 0.097). Bast et al. conducted a comprehensive study to compare COVID-19 patients in the pre-Delta and Delta waves. They revealed that patients in the Delta wave were more likely to be female and younger [26]. Stirrup et al. reported that women affected by the alpha strain had a higher risk of severe disease compared to men. Hence, the data found in all waves of the disease are confirmed in the ongoing study [27].

In an Iranian study, Amin et al. represented a significant difference between women and men infected with COVID-19, with men indicating higher rates of illness at the beginning. Nevertheless, as the pandemic progressed, the proportion of women gradually increased. Finally, more women were identified with COVID-19 during the fifth wave [21]. The most important risk factors between women and men are differences in the immune system, physiological factors, lifestyle, and sex hormones that lead to COVID-19 [24].

Although the proportion of total hospitalized cases and positive SARS-CoV-2 cases in the fifth (Delta) wave (39.3 and 68.3%, respectively) was more than twice as high as in the fourth (Alpha) (18.9 and 55.7%, respectively) and sixth (Omicron) waves (16.1 and 39%, respectively), the mortality rate in the Alpha wave (6.6 and 6.8%) was almost three times higher than in the Delta (2.7 and 2.7%, respectively) and Omicron (2 and 3.5%, respectively) waves. A downward trend in the mortality rate of patients was thus observed during the study period, while the highest mortality rate was recorded in the fourth wave of hospitalization.

This rate suggests that the Alpha variant resulted in more deaths than the original SARS-CoV-2. According to Lin’s meta-analysis, the risk of death was significantly higher in patients infected with the alpha variant than in patients infected with the original SARS-CoV-2. This is similar to our results [28]. On the other hand, despite the high percentage of deaths, patients in the fourth wave did not differ significantly from those in the fifth and sixth waves in terms of longer duration of hospitalization. Moreover, the present study found that the average number of comorbidities, mean age, and percentage of males were lower in the Delta wave than in the Alpha and Omicron waves.

Consistent with our report, the Delta variant had a higher mortality rate and severity. Studies in England [29], Denmark [30] and Iran [25] declare similar results. Twohig et al. indicated that the Delta variant causes severe disease compared to the Alpha variant [29]. Moreover, Zali et al. pointed out that the proportion of deaths in patients infected with the Alpha variant was higher than in patients infected with the Delta variant. However, the Delta variant (the second peak) was correlated with COVID-19 death risk, which is in agreement with the results of the current study [25]. Hence, the severity of COVID- 19 with the Omicron variant is likely milder (significantly lower morbidity and mortality) than with the Delta and Alpha variants detected by whole genome sequencing [31].

In addition, several factors have been identified as predictors of death and serious outcomes, including male gender, increasing age, and comorbidities such as hypertension, heart disease, diabetes, liver disease, and chronic KD [32]. On the other hand, Fano-Sizgorich et al. revealed that the Omicron variant was associated with the lowest risk of hospitalization and death in the crude analysis, consistent with greater transmissibility and lower disease severity.

However, during the Omicron wave, the number of hospitalized patients > 65 years increased and the proportion of deaths decreased in the fourth and fifth waves. Part of this lower severity is likely due to increased protection against the disease through previous immunity, vaccination, infection or a combination of these.

Another issue was the start of public coronavirus vaccination in Iran in May. Most people > 60 years old were vaccinated in August. Therefore, one of the reasons for the decrease in the death rate in the fifth and sixth waves can be seen in the general vaccination in Iran, especially among the elderly and high-risk individuals. However, the impact of vaccination on the reduction in the proportion of hospitalizations and deaths in the fifth and sixth waves is difficult to explain.

The results of the ongoing study showed that patients with the Delta variant had a higher viral load, which makes them more contagious. In addition, the results of this study demonstrated that there was no significant relationship between the duration of hospitalization and the Ct value. As for the distribution of deaths among COVID-19 patients, the frequency of deaths was higher in the group with a Ct of 9-20 than in the other groups and was statistically significant. Furthermore, the presence of a significant correlation between Ct and age was another interesting finding of the present study. The current study has certain limitations, such as the difficulty of interpreting clinical symptoms, the lack of CT scans for participants, and the possibility of errors during the pretest phase. Also, data regarding vaccination status and prior COVID infection was inaccessible. Moreover, in the ongoing study, the direct impact of the use of COVID-19 drugs (antivirals, monoclonal antibodies, and immunotherapy) on disease severity could not be assessed as no information on the patients was available. These factors could explain the differences in outcomes observed during the different COVID-19 waves after the general introduction of vaccination in Iran.

## Conclusion

To our knowledge, this largest study assessed the risk of hospitalization in confirmed cases in Iran during the Alpha, Delta, and Omicron waves. The highest number of hospitalized patients was in the fifth wave of COVID-19, while the sixth wave had the lowest number. According to the results of the present study, hospitalized patients in the fourth and fifth waves were younger, while hospitalized patients in the sixth wave were older. The incidence of hospitalization was higher in women than in men in all waves. Comorbidities were similar, and CVD, diabetes, KD, and hypertension were the main risk factors in all waves. The highest mortality rate was seen in the fourth wave, but the lowest in the fifth wave.

## Data Availability

All data obtained or analyzed as part of this study are included in this published article.

## Abbreviations

SARS-CoV-2: Severe Acute Respiratory Syndrome Coronavirus 2
WHO: World Health Organization
COVID-19: coronavirus disease 2019
IQR: interquartile range
rRT-PCR: Real-time reverse transcription polymerase chain reaction
CVD: Cardiovascular Disease
BD: Blood Disorders
Ct: Cycle threshold
